# Color routing at the nanoscale

**DOI:** 10.1038/s41377-019-0170-x

**Published:** 2019-06-26

**Authors:** Qing-Hua Xu

**Affiliations:** Department of Chemistry, National University of Singapore, 3 Science Drive 3, Singapore, 117543 Singapore

**Keywords:** Nanoparticles, Imaging and sensing

**Two types of color routing effects were observed in silver nanorods on the single particle level as a result of distinct far-field behaviors of different multipolar plasmon modes, which enables tunable color sorting with good wavelength selectivity.**

Photonics technologies are important for the development of modern information and communications technology, where light acts as a medium for carrying information and processing signals. Photonics allows high-speed and wide-bandwidth data handling beyond the limitations of conventional electronics technologies. The manipulation of light propagation is essential for photonics applications. Conventional methods of manipulating light propagation require the use of bulky optical elements. It is highly demanding and challenging to confine or route light on a miniaturized scale, especially on the nanoscale due to the diffraction limit of light^1^. The development of optical components for light manipulation on the nanoscale scale is critical for the development of photonics technology.

Noble metal nanoparticles are known to display the unique optical property of localized surface plasmon resonance, which arises from the collective oscillation of conduction band electrons induced by incident light irradiation^2^. This unique optical property allows the concentration of light into a subwavelength volume, which enables light manipulation at the nanoscale to overcome the diffraction limit^2–4^. Plasmonic nanostructures have been successfully demonstrated to manipulate light propagation, leading to phenomena such as light emission with controlled polarization and propagation direction, cloaking, and color routing^2–7^. Color routing, in which light of different colors is sorted into different directions, on the nanoscale is highly desired for all-optical communication devices and wavelength-encoded information^6,7^. However, nanoscale color routing is very challenging because it requires good wavelength selectivity and directionality at the same time.

Thus far, nanoscale color routing based on plasmon interference has been demonstrated in a few precisely designed nanostructures with multiple elements^6,7^. These previous schemes require careful design and control of plasmonic nanostructures with complex geometries that are prepared by sophisticated nanofabrication techniques. In a recent publication, Zhuo, Wang and coworkers reported two different color routing effects based on the high-order plasmon modes of single silver (Ag) nanorods on the single particle level^8^. These Ag nanorods can be conveniently prepared on a large scale with low cost using wet-chemical methods.

**Fig. 1:**
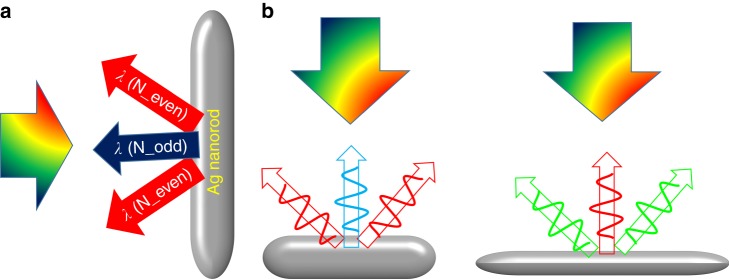
Color routing with a single Ag nanorod. **a** Different scattering directions for multipolar modes with odd and even symmetries. **b** Two different color routing phenomena with different patterns (red–blue–red and green–red–green) for Ag nanorods with different aspect ratios

In contrast to the designs in previous reports, this new scheme is based on longitudinal multipolar plasmon modes of Ag nanorods. These high-order plasmon modes were generally overlooked in previous studies because they are usually assumed to be “dark modes”, which are inaccessible by conventional far-field methods. However, as the length of Ag nanorods increases to comparable to the wavelength of visible light, the retardation effect will become important such that these multipolar plasmon modes can be conveniently accessible by conventional optical spectroscopy and imaging techniques. Consequently, Ag nanorods with high aspect ratios will display distinct far-field behaviors. The dark-field images of individual Ag nanorods of two different aspect ratios (390 × 64 nm and 567 × 66 nm) were found to display red–blue–red or green–red–green patterns, a new phenomenon that has never been previously reported. Numerical simulation confirmed that the physical origins of these patterns were the different far-field behaviors of longitudinal multipolar plasmon resonances with odd and even symmetries. Numerical simulations indicated that electromagnetic waves radiate along the central plane for modes with odd symmetry, while these waves radiate obliquely into the far field for multipolar modes with even symmetry (Fig. 1a). Depending on the aspect ratios of the Ag nanorods, light of different colors associated with multipolar modes of different symmetries will be scattered along different directions of the nanorods, exhibiting color routing effects. Two types of color routing phenomena with different patterns were experimentally observed at the single particle level (Fig. 1b).

The observed color routing phenomenon relies solely on the distinct far-field behaviors of the multipolar plasmon modes of long Ag nanorods with different symmetries. It does not require special geometries with careful size optimization, and therefore, the nanofabrication process is significantly simplified. The bandwidths of these multipolar plasmon modes are generally narrow, with their central wavelengths determined by the aspect ratio, which offers tunable color routing effects with good color selectivity. Although Ag nanorods were utilized as an illustrative example, this strategy can be easily extended to other plasmonic nanostructures, such as gold nanorods with high aspect ratios. In addition to color routing effects, carefully designed plasmonic nanostructures that support multipolar plasmon modes can also be employed for other light manipulation functions at the nanoscale, such as nanolasers and antennas. These studies will significantly increase the versatility of plasmonic devices and foster the development of nanoscale photonics technology and its practical application in different fields, such as nonlinear optics, optoelectronics, information technology, optical communication, sensing, and imaging.
